# Comparative analysis of generative LLMs for labeling entities in clinical notes

**DOI:** 10.1186/s44342-024-00036-x

**Published:** 2025-02-06

**Authors:** Rodrigo del Moral-González, Helena Gómez-Adorno, Orlando Ramos-Flores

**Affiliations:** 1 Posgrado en Ciencia e Ingeniería de la Computación, Universidad Nacional Autónoma de México, Circuito Escolar, Ciudad Universitaria, Coyoacán, 04510 Ciudad de México México; 2 Instituto de Investigaciones en Matemáticas Aplicadas y en Sistemas, Universidad Nacional Autónoma de México, Circuito Escolar, Ciudad Universitaria, Coyoacán, 04510 Ciudad de México México

**Keywords:** Zero-shot, Named entity recognition, Generative language models, Clinical domain, BLAH8

## Abstract

This paper evaluates and compares different fine-tuned variations of generative large language models (LLM) in the zero-shot named entity recognition (NER) task for the clinical domain. As part of the 8th Biomedical Linked Annotation Hackathon, we examined Llama 2 and Mistral models, including base versions and those that have been fine-tuned for code, chat, and instruction-following tasks. We assess both the number of correctly identified entities and the models’ ability to retrieve entities in structured formats. We used a publicly available set of clinical cases labeled with mentions of diseases, symptoms, and medical procedures for the evaluation. Results show that instruction fine-tuned models perform better than chat fine-tuned and base models in recognizing entities. It is also shown that models perform better when simple output structures are requested.

## Introduction

Manually extracting relevant information from electronic health records (EHRs) is expensive in terms of both resources and time. Most high-performing clinical named entity recognition (NER) methodologies require some form of predefined lexical data.

The field of NER has seen increasing research efforts with the creation of transformer architectures [6]. One of the main reasons for the interest in this specific task is that clinical text is very expensive to analyze using rule-based methods for its grammatical inconsistencies and rapidly changing lexicon. Other supervised machine learning methods have proven to be somewhat difficult to implement due to the scarcity of labeled corpora available.

Recently, we have also seen a rapidly increasing number of generative LLM models available for general and academic use. Many of these models have been released in different versions, each fine-tuned for specific purposes, and in various sizes depending on the number of parameters.

There have been some efforts to perform NER using generative LLMs. For example, [14] constructed a benchmark comprising twenty-two LLMs, evenly divided into eleven general and eleven medical models. This benchmark encompasses eleven downstream tasks, including named entity recognition (NER) for medical entities such as symptoms, medications, dosages, and procedures, using the BC5 and NCBI corpora. The authors evaluated these twenty-two LLMs using a zero-shot approach and compared the results to task-specific state-of-the-art (SOTA) models, which were fine-tuned on relevant downstream data and tasks. They adopted prompts used in current SOTA work to ensure fair evaluation for each task. The specific prompt for the NER task was derived from [2]. The proprietary model GPT-4 achieved the best zero-shot performance, with F1-scores of 71.3 on BC5 and 58.4 on NCBI. These scores, while impressive, fell short of the SOTA in NER, which has reached F1-scores of 90.0 on BC5 and 89.4 on NCBI.

In another 2024 study, [18] investigated the in-context learning (ICL) prompting method for biomedical NER across the I2B2, NCBI-disease, and BC2GM datasets. ICL involves presenting LLMs with example input-output pairs in a prompt to demonstrate the task. The authors enhanced this method with strategies like Translation between Augmented Natural Languages (TANL) and Data-Efficient Generative Models for Clinical Event Extraction (DICE), adapting them to biomedical NER. They also applied a retrieval-augmented generation (RAG) technique to incorporate definitions from the UMLS knowledge base into the prompts. Comparing zero-shot NER performance to fine-tuned state-of-the-art models, their method showed improved F1-scores across all datasets, achieving 53.1/62.8 (mention/token) on I2B2, 61.0/66.2 on NCBI-disease, and 52.1/58.4 on BC2GM.

Our research aims to fill a gap in exploring the capabilities of open-access generative models in a zero-shot setting for the NER task in the clinical domain. These generative models have shown promise in various NLP tasks using zero-shot settings (i.e., without using annotated corpora) [9]. Moreover, few related works investigated how the prompting strategies affect the output of sequence labeling.

The research questions that motivate our work are the following:

- How does the general vs. instruction-following fine-tuning affect zero-shot NER performance in the clinical domain?
- Can code-fine-tuned models improve the structural understanding needed for NER labeling tasks?
- How do open-access generative models compare in performance for zero-shot clinical NER tasks?

## Methods

### Dataset

We first compiled a set of clinical cases in the English language that have been labeled with mentions of diseases, symptoms, and medical procedures. These documents were sourced from the overlapping corpora of DisTEMIST [17], SympTEMIST [13], and MedProcNER [12]. The clinical cases from these datasets were initially written and labeled in Spanish, and their authors have released machine translations for English and other languages to support multilingual NER development. These translations are known as the silver-standard datasets and include both the texts and their corresponding labels.

These three datasets are derived from the same source documents, each containing labels for only one category of entities. Our first step was to compile these documents and merge their labels to create a unified label set encompassing all three entity categories. The datasets contain a total of 1000 common documents, although 250 lack disease annotations. Initially, we attempted to integrate the LivingNER [16] corpus, which includes mentions of living organisms; however, we found differences in its translation from the silver-standard texts of the other three datasets, limiting the overlapping dataset to only 90 documents. Consequently, we decided to use these 90 documents for our zero-shot experiments with generative LLMs, but we excluded the annotations from LivingNER for consistency with the training set. We used the remaining 910 documents to fine-tune encoder-based transformer models to compare the performance of our zero-shot approach. We also removed the overlap across entities of different categories, keeping only the longest entity span of each overlapping set.

The final version of this silver-standard dataset consists of 1000 clinical cases with an average length of 333 words. The documents in this dataset contain multiclass labels in the IOB tagging scheme [19], spanning a total of 21,867 mentions across diseases, symptoms, and medical procedures. Of these mentions, 2855 can be found in the test set and 19,012 in the train set. Table 1 provides a summary of label distribution across categories.

**Table 1:** Summary of entity mentions per source dataset

	DisTEMIST	SympTEMIST	MedProcNER
Train	4642	6569	7801
Test	809	1079	967
Total	5451	7648	8768

Number of entity mentions in 910 documents for the train set and 90 documents for the test set

### Open access large language models

The Llama 2 model [21] has a version that has been fine-tuned to chat with humans, using a dataset of around 27,000 real prompt-answer pairs. There is also a code fine-tuned version of the Llama 2 model [20], which is called Code-Llama and has further training on a code-heavy corpus.

On the other hand, the Mistral language model [7] has a version fine-tuned for following instructions, which its authors have compared to the Llama 2 chat version. Additionally, Mistral has a version called Mixtral [8], which uses a Sparse Mixture of Experts (SMoE) architecture. In this architecture, the number of feed-forward layers, referred to as “experts,” is scaled and added to the output of the model. Then, a gating mechanism is used to decide which of the experts will process the output. This architecture is meant to improve the generation capabilities of the model while barely increasing the computational cost.

### Experimental settings

Our study addresses several key questions to explore the potential of open-access generative models in zero-shot clinical NER. First, we aim to examine how instruction-following fine-tuning affects model performance in this context, comparing instruction-tuned versions (such as mistral-7b-instruct-v0.2 and mixtral-8x7b-instruct-v0.1) with their non-instruction-tuned counterpart mistral-7b-v0.1. This comparison aims to assess whether models optimized for prompt adherence can achieve higher accuracy in complex, structured tasks like clinical NER, where precise guidelines are often required. Additionally, we investigate whether code-fine-tuned models, such as codellama-7b-instruct, offer advantages in handling structured sequence labeling. Given that code-fine-tuning emphasizes syntactic and structured inputs, we hypothesize that these models may exhibit improved boundary recognition and entity categorization in NER tasks. In summary, our study evaluates the effectiveness six open-access generative models: llama-2-7b, llama-2-7b-chat, codellama-7b-instruct, mistral-7b-v0.1, mistral-7b-instruct-v0.2, and mixtral-8x7b-instruct-v0.1 a in zero-shot settings for clinical NER to establish a comparative baseline.

For these experiments, we employed models with identical parameters, and developed three prompt variations, each providing increasingly detailed instructions to the models. We performed these experiments using an NVIDIA A40 GPU with 48GB of RAM, from a private cloud computing service.

The exact text of these prompts can be found in Table 2. In the first prompt variation, we described the objective task and defined the categories of entities to be extracted, but we did not specify any output format. This first prompt variation was proposed to evaluate how well models can understand the NER task and produce coherent free-structured outputs. In the second variation, we did not only detail the task and entity categories, presenting them in a code-like list format, but also provided an example output formatted as a Python dictionary. With this variation, we want to observe how well the different models can produce a valid output using a simple structure. Importantly, we did not suggest that these formats were tied to a specific programming language. Finally, in the third variation, we specified to the models that they had to generate the output in a more complex structured JSON-like format. We also set inside the prompt a complete example of the desired output so the model had an example of the output structure.

**Table 2:** Prompt variations given to the LLMs

Prompt	Prompt
Variation 1	“You are an expert in labeling clinical notes with mentions of diseases, symptoms, and medical procedures. Extract the mentions of diseases, symptoms, and medical procedures from the following clinical note: **clinical text**”
Variation 2	“You are an expert in labeling clinical notes with mentions of diseases, symptoms, and medical procedures. Please identify the entities of types [disease, symptom, medical procedure] in the following clinical document. The output must be formatted like this: {diseases: [list of diseases], symptoms: [list of symptoms], medical procedures: [list of medical procedures]} Document: **clinical text**”
Variation 3	“You are an expert in labeling clinical notes with mentions of diseases, symptoms, and medical procedures. Analyze the following clinical note and identify all mentions of diseases, symptoms, and medical procedures. Provide the annotations in a structured JSON format. Each identified entity should be categorized as either a disease, symptom, or medical procedure. Example JSON format for annotations (Note: Replace “entity1”, “entity2”, etc., with actual entity names: { “annotations”: [ { “entity”: “entity1”, “category”: “disease” }, { “entity”: “entity2”, “category”: “symptom” }, { “entity”: “entity3”, “category”: “medical procedure” } ... more entities ] }. Now, analyze this clinical note: **clinical text**”

The **clinical text** legend was substituted with the actual clinical cases from the dataset

We defined two evaluation strategies to assess the output of the models for the zero-shot NER task:

- The first evaluation strategy focuses solely on whether the model is able to recognize the entities that are present in the silver-standard annotations. We iterate over each term in the silver-standard, and for each term, we identify if a mention exists in the LLM output. If there is a complete or partial match, we tag all the occurrences of the term in the clinical case document using the B or I tags of the IOB tagging scheme without considering the entity category. The rest of the words in the document that are not identified as an entity are labeled with the O tag. With this strategy, we aim to assess the ability of the model to detect entities regardless of the format of the output requested in the prompts.
- For the second strategy, we use regular expressions to identify the exact structures that have been requested to the LLM. This strategy is designed to evaluate the prompt variations 2 and 3. In this way, we first evaluate whether the model accurately produces the required formats―a Python dictionary or a JSON-valid output. Then, we determine if the model populates these structures using entities that have been correctly detected and extracted. In this strategy, we first iterate over each entity category (symptoms, procedures, and diseases) produced by the LLM output; then, for each entity in the category, we identify if the entity appears (complete or partial) in the clinical case document. If the entity appears in the document, we tag all the occurrences of the term using the B or I tags according to the specific category. Essentially, this second strategy aims to evaluate the LLMs’ performance on the NER task when a specific structure is requested.

To evaluate the open-access generative LLMs, we queried each of the previously mentioned models with each of the three prompts for each clinical case document. We assessed their outputs using our two predefined evaluation strategies.

Finally, we compared the zero-shot performance of the generative LLMs against fine-tuned encoder-based neural models, both based on transformer architectures, to assess their relative performance. Initially, we evaluated three base models: BERT [4], RoBERTa [15], and XLM-RoBERTa [3]. Following this, we expanded our evaluation to include models pre-trained specifically on biomedical data, such as BIO-BERT [11], Clinical-BERT [1], and specialized RoBERTa variants like BIOMED-RoBERTa [5] and Clinical-XLM-RoBERTa [10]. Fine-tuning experiments were conducted using two NVIDIA RTX A5000 GPUs, with 25 GB of RAM. Models were trained for 3 and 5 epochs, with the best results obtained after five epochs and summarized in Table 6.

## Results

For the first evaluation strategy, we generated IOB labels for the original documents, using the entities that are present in the LLM outputs as described in the previous section. Then, we calculated precision, recall, and F1 scores using these IOB labels. The obtained metrics are presented in Table 3.

**Table 3:** Precision, recall, and F1 metrics for entities identified in the outputs of the LLMs, using the first evaluation strategy

	Variation 1			Variation 2			Variation 3		
Model	P	R	F	P	R	F	P	R	F
llama-2-7b	0.986	0.084	0.156	0.907	0.023	0.046	0.967	0.052	0.099
llama-2-7b-chat	0.971	0.182	0.306	0.952	0.191	0.318	0.957	0.145	0.252
codellama-7b-instruct	0.971	0.383	0.549	0.961	0.220	0.358	0.967	0.191	0.318
mistral-7b-v0.1	0.960	0.043	0.083	0.951	0.046	0.088	0.941	0.038	0.074
mistral-7b-instruct-v0.2	0.966	0.222	0.361	0.955	0.267	0.418	0.929	0.118	0.209
mixtral-8x7b-instruct-v0.1	0.974	0.428	**0.595**	0.974	0.365	**0.531**	0.958	0.193	**0.321**

The highest F1-scores for each prompt variation are highlighted in the table

For the second evaluation strategy, after parsing the outputs according to the Python dictionary and JSON formats, we obtain the IOB labels using all the entities that were correctly parsed. Finally, we calculated precision, recall, and F1 scores. The results by LLM and entity category are presented in Table 4. This second evaluation strategy was specifically applied to responses from prompt variations 2 and 3.

**Table 4:** Precision, recall, and F1 metrics for entities present in the formatted outputs of the LLMs, using the second evaluation strategy and expanded by entity category

		Variation 2			Variation 3		
Model	Category	P	R	F1	P	R	F1
llama-2-7b	DISEASE	0.500	0.008	0.015	0.333	0.003	0.005
	PROCEDURE	0.000	0.000	0.000	0.500	0.001	0.003
	SYMPTOM	0.667	0.004	0.008	0.000	0.000	0.000
	micro avg	0.556	0.003	0.006	0.222	0.001	0.003
llama-2-7b-chat	DISEASE	0.349	0.055	0.096	0.500	0.040	0.075
	PROCEDURE	0.000	0.000	0.000	0.323	0.015	0.028
	SYMPTOM	0.494	0.080	0.138	0.472	0.033	0.062
	micro avg	0.432	0.040	0.073	0.434	0.027	0.051
codellama-7b-instruct	DISEASE	0.413	0.096	0.155	0.000	0.000	0.000
	PROCEDURE	0.000	0.000	0.000	0.000	0.000	0.000
	SYMPTOM	0.560	0.210	0.305	0.000	0.000	0.000
	micro avg	0.512	0.091	0.155	0.000	0.000	0.000
mistral-7b-v0.1	DISEASE	0.167	0.003	0.005	0.462	0.015	0.029
	PROCEDURE	0.667	0.003	0.006	0.250	0.001	0.003
	SYMPTOM	0.462	0.012	0.023	0.400	0.004	0.008
	micro avg	0.409	0.006	0.011	0.409	0.006	0.011
mistral-7b-instruct-v0.2	DISEASE	0.467	0.141	0.217	0.571	0.101	0.171
	PROCEDURE	0.000	0.000	0.000	0.421	0.012	0.023
	SYMPTOM	0.517	0.208	0.297	0.390	0.080	0.133
	micro avg	0.498	0.102	0.170	0.459	0.056	0.100
mixtral-8x7b-instruct-v0.1	DISEASE	0.463	0.189	0.268	0.372	0.146	0.210
	PROCEDURE	0.429	0.031	0.058	0.319	0.087	0.137
	SYMPTOM	0.434	0.237	0.307	0.398	0.157	0.225
	micro avg	0.443	0.137	**0.209**	0.363	0.124	**0.185**

The highest F1-scores for each prompt variation are highlighted in the table

Additionally, to separately evaluate the ability of the models to produce specific formats, we also calculated the ratio of responses that correctly presented at least one entity in the requested format. Table 5 shows these results.

**Table 5:** Ratio of documents with correctly parsed entities

Model	Variation 2	Variation 3
llama-2-7b	0.16	0.16
llama-2-7b-chat	0.59	0.34
codellama-7b-instruct	**0.99**	0.16
mistral-7b-v0.1	0.13	0.11
mistral-7b-instruct-v0.2	0.98	**0.99**
mixtral-8x7b-instruct-v0.1	0.86	0.98

The highest scores for each prompt variation are highlighted in the table

Finally, Table 6 shows the performance of some encoder-based neural models that we fine-tuned for five epochs using the 910 clinical cases of the training subset from our unified silver-standard dataset.

**Table 6:** Micro-average precision, recall, and F1-score for entities identified by the fine-tuned encoder-based transformer models

Transformer LM	*P*	*R*	F1
BERT	0.511	0.583	0.545
RoBERTa	0.532	0.616	0.570
XLM-RoBERTa	0.512	0.585	0.546
BIO-BERT	0.541	0.622	**0.579**
BIO-Clinical-BERT	0.519	0.600	0.557
BIOMED-RoBERTa	0.527	0.617	0.569
Clinical-XLM-RoBERTa	0.547	0.597	0.571

The highest F1-score is highlighted in the table

Table 7 presents examples of outputs generated by the LLM alongside their respective silver-standard annotations.

**Table 7:** Output examples resulted from the three prompt variations for the same document on the mixtral-8x7b-instruct-v0.1

Prompt	Output
Variation 1	1. Diseases:
	- Fever
	- Sub-Saharan origin (assumed geographical disease association)
	- Eosinophilia
	- Renal hydatid disease (also known as hydatid cyst or echinococcosis)
	2. Symptoms:
	- Sweating
	- Fever
	- Left lumbar pain
	- Heaviness in the left flank
	- Dysuria (painful urination)
Variation 2	Here are the entities of types disease, symptom, and medical procedure identified in the clinical document:
	* Diseases: [renal hydatid disease;]
	* Symptoms: [sweating; fever; left lumbar pain; heaviness in the left flank; dysuria; occasional fever; voiding symptoms; acceptable urinary stream; night-time voiding once and daytime voiding every 3–4 hours with a feeling of complete emptiness]
	* Medical procedures: [physical examination; rectal examination; complementary tests; X-ray of the genito-urinary tract; renal ultrasound; abdominopelvic CT scan; echinococcus/hemag antibody serology; MRI; surgery; course of Albendazole; left nephroureterectomy] Note that some of the symptoms are mentioned more than once in the document, but I have only included each symptom once in the list. Also, some of the medical procedures are not specific to this case, and could be part of a general clinical workup, but I have included them for completeness.
Variation 3	{ annotations:[ {entity: sub-Saharan origin, category: not applicable}, {entity:sweating and fever} ]
Silver-standard	{diseases: [lesions, cystic lesions, hydatid cyst, allergies, DM], symptoms: [colic, urinary stream, urgency, mass effect in the left flank, haematuria, Normal biochemistry, sweating, shortness of breath, eosinophilia, voiding symptoms, lumbar pain, occasional fever, normal haemogram, abdominal mass, Echinococcus/Hemag antibody serology was requested, which was POSITIVE, dribbling, feeling of complete emptiness, negative urine analysis, fever, asymptomatic, dysuria, heaviness in the left flank], medical procedures: [left flank on deep palpation, Renal ultrasound, Abdominopelvic CT scan, surgical treatment, complete left nephroureterectomy, MRI, Rectal examination, Physical examination, surgery]}

For demonstration purposes, the silver-standard output uses the format requested by the second prompt variation

## Discussion

In the first evaluation strategy, the precision is consistently high across all models. Since the entity boundaries are not defined in the output of the LLM, these results only indicate that the models can correctly identify and transcribe the entities that are present in the clinical case. The generally low recall value shows the proportion of entities that the models managed to identify. One primary reason for these values may be the inherent limitations of using generative models in a zero-shot setting for the NER task, as these models lack task-specific fine-tuning, which could lead to under-detection of entities not explicitly emphasized in the prompt. Even though the recall is generally rather low, we can see that instruction models are better at retrieving information. Also, we observed that the models trained on code-heavy corpora have a slight advantage over the others. We also notice that as the complexity of the instruction and the formatting of the output increases, the model’s focus shifts away from the primary task, resulting in fewer identified entities.

The results for the second evaluation strategy presented in Table 4 show that precision is generally better for prompt variation 2, which requests a simpler format for the output. This is consistent with the ratio of documents with valid outputs (see Table 5) and provides insight into using generative LLMs for NER at the entity level. The task benefits from simple output structures, which can then be aligned with the texts using post-processing algorithms to obtain valid mention-level IOB labels. When comparing only the results from the instruction fine-tuned models, mixtral-8x7b-instruct-v0.1 shows a stronger performance compared to codellama-7b-instruct. Our explanation for this difference is that the mixtral model is a more general-purpose LLM that has the advantage of its mixture of experts architecture, whereas codellama-7b-instruct has a simpler architecture and specializes primarily in code-related tasks. Regarding category-level performance, identifying diseases and symptoms proves easier across all models, while medical procedure extraction tends to perform less accurately. We hypothesize that this may be due to the broader scope of the concept of medical procedures, compared to diseases or symptoms. Still, the mixtral models show improved performance, likely due to their mixture-of-experts architecture

The results presented in Table 5 aim to demonstrate the ability of the LLM to produce specific outputs (with fewer hallucinations). In this table, we observe only that instruction models are better at producing specific outputs. Interestingly, the codellama model could not consistently produce the JSON-like structure with prompt variation 3. By analyzing the outputs of this specific model and prompt combination, it appears that the model attempts to explain and even diagnose the clinical cases, thus forgetting to produce the structured output. Although this model was tested for safety regarding truthfulness, toxicity, bias, and harmful code generation, there seems to have been no specific tuning to prevent the model from outputting health diagnosis-related information [20]. In Llama 2, there was some feedback training after suggestions from a group of risk and security advisors, so it is possible that further fine-tuning caused the model to drift away from that training. The Llama 2 model presented outputs where it refuses to generate a response since the prompt contains personal data and mentions of injuries.

Finally, for the performance comparison with encoder-based models, as shown in Table 6, models pre-trained on domain-specific biomedical data consistently outperformed those trained on general domain datasets. These results demonstrate the value of domain adaptation for biomedical tasks. Notably, BIO-BERT achieved the highest F1-score. This performance level was comparable to results obtained with the mixtral-8x7b-instruct-v0.1 model under the zero-shot setting, particularly when using the prompt 1 variation in our the initial evaluation strategy. This comparison provides insight into the efficacy of specialized fine-tuned models relative to large, general-purpose LLMs, underscoring the advantage of domain-specific pretraining in achieving optimal performance.

## Limitations

The non-deterministic nature of LLMs can impact the reliability of the results presented in this work because the same inputs can generate different results. LLMs provide flexibility and the ability to perform NER tasks without the need for annotated training data. However, their outputs are generally less reliable and consistent than those of traditional, fine-tuned discriminative models.

## Ethics statement

This research uses publicly available datasets. However, when working with clinical data, it is important to consider issues like data privacy and ethical use. Deploying NER models in real-world clinical settings requires detailed validation to ensure patient confidentiality and adherence to data protection regulations.

## Conclusion

This study evaluated and compared different fine-tuned variations of generative LLMs for zero-shot named entity recognition (NER) in the clinical domain. Our findings indicate that instruction fine-tuned models generally perform better in identifying entities.

The analysis of prompt variations shows that simpler output structures improve the performance of the models in the NER task. It was also found that fine-tuned models might lose some adjustments made for safety reasons, particularly in preventing the generation of unwanted diagnostic information.

Overall, models fine-tuned to follow instructions show superior performance in zero-shot biomedical NER tasks when using simple output structures. This suggests their potential to complement other supervised NER systems that may lack a large training corpus.

## Data Availability

Data is provided within the manuscript or supplementary information files.
